# The *Arabidopsis* E3 ubiquitin ligase PUB13 synergistically interacts with BON1 to regulate plant flowering and immunity

**DOI:** 10.3389/fpls.2025.1585221

**Published:** 2025-06-02

**Authors:** Jiani Yue, Xiuwei Zou, Yu Peng, Sujun Pan, Cailin Hu, Bing Wang, Liangying Dai, Wei Li

**Affiliations:** ^1^ College of Plant Protection, Hunan Agricultural University, Changsha, China; ^2^ Technology Center, China Tobacco Hunan Industrial Co., Ltd., Changsha, China; ^3^ College of Agronomy, Hunan Agricultural University, Changsha, China

**Keywords:** PUB13, E3 ubiquitin ligase, BON1, flowering, resistance, immunity

## Abstract

The ubiquitination pathway is extensively involved in the regulation of plant biological processes, such as growth, development, and disease resistance, among others. Our previous study showed that the *Arabidopsis* U-box protein PUB13 regulates plant cell death, immunity, and development. Here, we report that the E3 ubiquitin ligase activity of PUB13 is required for its regulation of the plant size, flowering time, and immunity based on the analysis of the biological processes on the PUB13 enzyme activity loss mutant. Furthermore, we identified the copine protein BON1 interacting with PUB13, which was ubiquitinated by PUB13. Interestingly, the knockout of *BON1* in the *pub13* mutant further strengthens its phenotypes of retarded growth and early flowering. In addition, the knockout of *BON1* further enhanced the resistance of *pub13* to the biotrophic pathogen. In contrast, the *pub13bon1* double mutant was more susceptible to the necrotrophic pathogen compared with the *pub13* and *bon1* single mutants. The synergistic effect between PUB13 and BON1 was also observed in the regulation of pathogen-associated molecular pattern-triggered immunity (PTI). These results indicate that the E3 ubiquitin ligase activity is required for PUB13 regulating biological functions and that BON1 synergistically interacts with PUB13 to regulate plant growth, flowering, and immunity in *Arabidopsis*.

## 1 Introduction

Protein is constantly produced and degraded during the whole life cycle of an organism. One of the most important protein degradation strategies in eukaryotic cells is the ubiquitin–26S proteasome system (UPS). However, not all proteins are degraded by the UPS after ubiquitination: except for poly-ubiquitination, mono-ubiquitinated and multi-ubiquitinated proteins are not degraded by the UPS, but can alter localization, affect the protein–protein interaction, or regulate the protein activity (Mukhopadhyay and Riezman, 2007; Ye and Rape, 2009). The UPS involves the sequential action of three enzymes, namely, E1 (the ubiquitin-activating enzyme), E2 (the ubiquitin-conjugating enzyme), and E3 (the ubiquitin ligase enzyme), to ultimately ligate one or more ubiquitin molecules to specific target proteins (Sun et al., 2019; Xu and Xue, 2019). Plant U-box proteins (PUBs) are the earliest discovered E3 ubiquitin ligases, and their U-box domain is structurally conserved (Koegl et al., 1999; Azevedo et al., 2001). In *Arabidopsis*, the largest class of PUBs includes the Armadillo (ARM) repeat domain behind the U-box domain (Mudgil et al., 2004). The ARM domain generally exists in eukaryotes with three to eight repeats and plays a role mainly in protein–protein interaction (Peifer et al., 1994; Samuel et al., 2006).

E3 ubiquitin ligases play an important role in plant growth and development. For example, the rice U-box/ARM protein OsPUB15 regulates rice growth during the seedling stage [10]. With the knockout of *OsPUB15*, the plant primary roots were inhibited during germination, and the shoot development of the *pub15* mutant was also significantly slower than that of the wild type (Park et al., 2011). In addition, the rice PUB protein Spotted Leaf 11 (SPL11) with ubiquitin ligase E3 activity mono-ubiquitinated and repressed SPL11-interacting Protein 1 (SPIN1) to positively regulate the flowering time (Vega-Sánchez et al., 2008). In contrast, *Arabidopsis* PUB13, an ortholog of SPL11, negatively regulated the flowering time (Li et al., 2012a). Under long-day conditions, the *pub13* mutant exhibited a significant early flowering phenotype, with a decrease in the transcription level of the negative flowering regulator *FLOWERING LOCUS C* (*FLC*), while the transcription levels of the positive regulators *FLOWERING LOCUS T* (*FT*) and *SUPPRESSOR OF OVEREXPRESSION OF CONSTANS1* (*SOC1*) were increased (Lu et al., 2011; Li et al., 2012a; Antignani et al., 2015).

E3 ubiquitin ligases are also involved in plant disease resistance. For example, SPL11 negatively regulates the plant defense against the rice blast pathogen (*Magnaporthe grisea*) and the bacterial blight pathogen (*Xanthomonas oryzae* pv. *oryzae*). Similarly, the SPL11 orthologs PUB12 and PUB13 in *Arabidopsis* play an important role in the regulation of plant pathogen-associated molecular pattern (PAMP)-triggered immunity (PTI) (Lu et al., 2011; Li et al., 2012a; Antignani et al., 2015). Upon stimulation with flagellin, the receptor kinase BAK1 phosphorylated PUB12 and PUB13, thereby activating the ubiquitination and UPS degradation of the flagellin receptor FLS2 by PUB12 and PUB13. This signal transduction ultimately led to the negative regulation of FLS2 by PUB12 and PUB13 of the plant PTI (Lu et al., 2011; O’Neill and , 2011; Zhou et al., 2015). Similarly, the expression of the *Arabidopsis* E3 ubiquitin ligases PUB22/PUB23/PUB24 can be induced by flagellin, and the mutants *pub22*, *pub23*, and *pub24* significantly improve plant resistance to pathogens (Trujillo et al., 2008; Stegmann et al., 2012; Chen et al., 2014; Wang et al., 2020). Furthermore, both *Arabidopsis* PUB20 and PUB21 negatively regulate plant innate immunity, negatively regulating plant resistance to the bacterial pathogen *Pseudomonas syringae* pv. *tomato* DC3000 (*Pst* DC3000) (Heise et al., 2002; Yee and Goring, 2009; Yi et al., 2024). In addition, PUB25 and PUB26 negatively regulate plant immunity by specifically targeting the non-activated BIK1 for 26S-UPS degradation in *Arabidopsis* (Wang et al., 2018). Interestingly, the E3 ubiquitin ligase PUB CMPG1 is required for cell death activation in *Nicotiana benthamiana* through Cf-9/Avr9, Cf-4/Avr4, Pto/AvrPto, or a cellulose-binding elicitor lectin (CBEL) (Gilroy et al., 2011). In addition, the orthologous tobacco ACRE276, the tomato ACRE276, and the *Arabidopsis* PUB17 are PUBs, and all of them regulate the plant effector-triggered immunity (ETI) (Yang et al., 2006; He et al., 2015). The transiently expressed *Arabidopsis* PUB17 can complement the silencing of ACRE276 in tobacco, restoring the Cf-9/Avr9- and Cf-4/Avr4-mediated hypersensitive response (HR); however, PUB17 lacking E3 ubiquitin ligase activity is not able to complement the silenced ACRE276, indicating that E3 ubiquitin ligase activity plays a key role in ETI signaling (Yang et al., 2006).

Copines are a group of Ca^2+^-dependent phospholipid-binding proteins that are widely distributed in eukaryotes (Warner et al., 2013; Tang et al., 2021). There are two calcium-dependent phospholipid-binding C2 domains at the amino (N)-terminus of copine proteins and a putative protein–protein interaction vWA (von Willebrand A) domain at the carboxyl (C)-terminus (Rizo and Südhof, 1998; Whittaker and Hynes, 2002). The ubiquitous conservation of copines among eukaryotes suggests that they play important roles in common biological pathways, which is supported by emerging studies that have demonstrated their roles in plant development, defense, and stress responses (Yang and Hua, 2004; Yang et al., 2006; Gou et al., 2015; Yang et al., 2017; Yin et al., 2018; Chen et al., 2020).

*Arabidopsis* BON1 is a copine protein, and the *bon1* mutant has shown inhibited growth at low temperature (22°C), while the phenotype could be recovered at 28°C (Hua et al., 2001). In addition, BON1 Associated Protein 1 (BAP1) has similar functions in growth regulation. Under 22°C growth conditions, the overexpression of BAP1 can restore the *bon1* mutant to the wild-type phenotype (Hua et al., 2001; Li et al., 2010). This suggests that BON1 is necessary for plants to maintain normal growth at low temperatures. Moreover, the *Arabidopsis* BON1, BON2, and BON3 exhibit a functional overlap, and they are necessary for the normal growth of *Arabidopsis* (Yang et al., 2006).

On the other hand, BON1, BON2, and BON3 have been reported to negatively regulate plant disease resistance (Yang and Hua, 2004; Yang et al., 2006). The *bon1* mutant shows enhanced disease resistance to the bacterial pathogen *Pst* DC3000 and the oomycete pathogen *Hyaloperonospora parasitica*, largely resulting from the upregulation of the plant immune receptor NLR (NOD-like receptor) gene *SNC1* (Yang and Hua, 2004). Furthermore, at least four R-like genes are induced in *bon1* and *bon3*, indicating that BON1 and BON3 negatively regulate disease resistance mediated by R-like genes (Li et al., 2009). Similarly, both *OsBON1* and *OsBON3* are negative regulators of disease resistance in rice (Yin et al., 2018).

In this study, we show that the E3 ubiquitin ligase activity of PUB13 is required for the regulation of plant growth, development, and disease resistance. In addition, a PUB13 interacting protein, BON1, was screened using yeast two-hybrid assay, which was found to be ubiquitinated by PUB13. Moreover, our data indicate that BON1 enhances the function of PUB13 on the growth, flowering, and disease resistance.

## 2 Materials and methods

### 2.1 Plants and growth conditions

The *Arabidopsis* (*Arabidopsis thaliana*) wild type (Col-0), *pub13* (Li et al., 2012a), *PUB13^V273R^/pub13*, *PUB13/pub13*, *bon1* (Hua et al., 2001), and *pub13(−/−)bon1(+/−)* were used in this study. The *pub13(−/−)bon1(+/−)* mutant was constructed by genetic crosses. PUB13 and PUB13^V273R^, driven by a native promoter 2 kb upstream of the *PUB13* start codon, were constructed into the binary vector pCAMMBIA1300. Subsequently, the *Agrobacterium tumefaciens*-mediated flower-dipping method of transformation was performed according to a standard protocol to obtain *PUB13^V273R^/pub13* and *PUB13/pub13* genetic plants. *Arabidopsis* plants were grown in nutrient soil at 22°C, 75% relative humidity (RH), and 8-h light/16-h dark as short day (regular condition) or 16-h light/8-h dark as long day in growth chambers.

### 2.2 Pathogen inoculation

Plants (4 weeks old) were inoculated with different pathogens at long-day conditions. *Pseudomonas syringae* pv. *maculicola* ES4326 and *P. syringae* pv. *tomato* DC3000 were sprayed on the plants at 1 × 10^8^ CFU ml^−1^ for disease symptoms or injected at 5 × 10^5^ CFU ml^−1^ for the bacterial growth assay according to (Li et al., 2012a). For *Botrytis cinerea* inoculation, the fungus was cultured on potato dextrose agar (PDA) media for 2 weeks at 24°C with a 12-h photoperiod. The fungal culture was washed with distilled water and filtered using sterile gauze. Subsequently, the concentration was adjusted to 3 × 10^4^ conidia ml^−1^. The detached rosette leaves were placed in Petri dishes containing 0.8% agar, 5 μl of the conidia suspension was dropped onto the leaf surface, and the Petri dishes were sealed. The inoculated leaves were incubated at 22°C with a 12-h photoperiod. The diameter of the lesion was measured at 48 h post-inoculation.

### 2.3 Yeast two-hybrid assay

The full-length coding sequence (CDS) of the *PUB13^V273R^* and *BON1*/prey library was individually inserted into the BD vector (pDBLeu) and the AD vector (pPC86), respectively. The pDBLeu and pPC86 empty vectors were used as negative controls for the screening and confirmation in this assay. The constructs or the corresponding empty vector (control) was transformed into the MATα yeast strain MaV203. The transformed yeast cells were plated on SD/-Leu/-Trp and SD/-Leu/-Trp/-His dropout media and cultured at 30°C for 2–5 days. Positive yeast colonies with successful transformation were selected for the X-gal assay on YPDA medium (ProQuest™ Two-Hybrid System with Gateway™ Technology, Invitrogen, Carlsbad, CA, USA) (Li et al., 2012b).

### 2.4 GST pull-down

The full-length CDS of *PUB13* and *BON1* was cloned into the pGEX-6p-1 and pMAL-c4x vectors, respectively. The purified PUB13:MBP and BON1:GST fusion proteins were mixed, and 60 µl of pre-rinsed glutathione Sepharose beads (Promega, Madison, WI, USA) was co-incubated with the protein mixture for 4 h at 4°C. The beads were then washed five times with 1× TBST buffer. Finally, the protein was eluted from the beads and was used for the Western blot assay. The BON1 and PUB13 proteins were detected with anti-MBP and anti-GST antibodies, respectively, in the Western blot assay, with the antibodies diluted to 1/1,500.

### 2.5 E3 ubiquitination assay

The full-length CDS of PUB13 was cloned into the pGEX-6P-1::GST vector, while BON1 was cloned into the pET-28a::myc vector. The fusion protein was expressed in *Escherichia coli* BL21, with 1.0 μg of PUB13/V273R-GST and BON1-myc used for each reaction. The wheat (*Triticum aestivum*) E1 (GI:136632; approximately 40.0 ng) and the human E2 (UBCH5b; approximately 40.0 ng) were used in the *in vitro* E3 ligase activity assays as described (Xie et al., 2002). The reaction samples were separated by 10% SDS-PAGE and detected by Western blot using an anti-myc antibody.

### 2.6 Luciferase complementation imaging

The full-length CDS of *PUB13* was cloned into the pCAMBIA-NLuc vector, while the full-length CDS of BON1 was cloned into the pCAMBIA-CLuc vector. Subsequently, they were transferred into the *Agrobacterium* strain GV3101 and the cells cultured at 28°C until ∽2.0 optical density (OD). The cells were harvested, resuspended, and then incubated with 10 mM MES solution containing 0.2 mM acetosyringone for 3 h at 28°C. The cells were harvested and resuspended in MES solution to yield 1.0 OD suspension. The NLuc and CLuc vectors were co-infiltrated into *N. benthamiana* leaves, and the RNAi suppressor P19 vector with 0.5 OD was also infiltrated. After storage at 26°C (dark) for 48 h, the leaves were sprayed with fluorescein potassium (CellGro, Manassas, VA, USA) and photographed using a chemiluminescence imaging system (Bio-Rad, Hercules, CA, USA).

### 2.7 Measurement of ROS accumulation

A luminol-based assay was employed to monitor the production of reactive oxygen species (ROS) (Li et al., 2021). Leaf discs were punched from the adult plant before flowering and soaked in sterilized distilled water in the dark overnight. Three leaf discs were placed in a 1.5-ml tube containing 1 μl of 100 nM flg22 or sterilized distilled water as a control, 1 μl of 10 μg/ml horseradish peroxidase (HRP), and 100 μl of 0.2 mM chemiluminescent probe L-012. Luminescence was detected immediately at 1-min intervals for 20 min with the Glomax 20/20 Luminometer. Each sample and treatment condition was repeated three times.

### 2.8 RNA extraction and RT-qPCR analysis

Total RNA was extracted using the TRIzol reagent (TransGen, Beijing, China) and was treated with a gDNA remover. Thereafter, the RNA was reverse-transcribed to cDNA using a cDNA synthesis kit (TransGen, Beijing, China). The cDNA was then diluted to 20 ng/μl before use as a template for real-time quantitative PCR (RT-qPCR). Three replicates were carried out per sample. Subsequently, RT-qPCR was conducted with the Bio-Rad CFX96 PCR amplifier. Three independent repeats were performed.

## 3 Results

### 3.1 The E3 ubiquitin ligase activity of PUB13 is required for regulating plant growth

**Figure 1:**
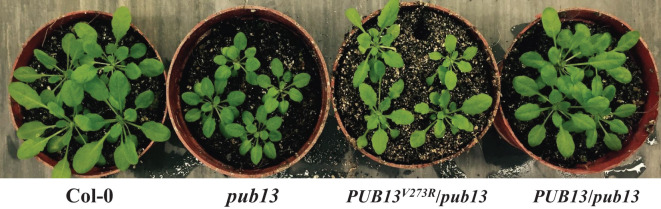
The E3 Ubiquitin Ligase Activity of PUB13 Is Required for Regulating Plant Growth. The indicated plants were 45 days old and grown under short day conditions.

PUB13 functions as a regulator of flowering, cell death, and immunity in *Arabidopsis* [12,14], and it is an active E3 ubiquitin ligase. The 273rd amino acid valine (V273) in the U-box domain is highly conserved and critical for PUBs. In this research, to determine the biological functions of the E3 ubiquitin ligase activity of PUB13, we created the *PUB13^V273R^* construct by substituting V273 to arginine, the mutation that suppresses the E3 ubiquitin ligase activity of PUB13 (Li et al., 2012a). The *PUB13^V273R^* construct was transformed into *pub13* plant (*PUB13^V273R^/pub13*), with the wild-type *PUB13* gene also transformed into *pub13* (*PUB13/pub13*). When the plants were 45 days old, the plant size of the *pub13* mutant was observed to be noticeably smaller than the wild-type Col-0 under short-day conditions (**Figure 1**). However, the complemented plant *PUB13/pub13* recovered to the wild-type level. Interestingly, the plant size of *PUB13^V273R^/pub13* was still smaller than the wild type (**Figure 1**). Subsequently, the rosette diameter, the leaf petiole length, and the leaf lamina length of Col-0, *pub13*, *PUB13^V273R^/pub13*, and *PUB13/pub13* were compared. The numerical results of these parameters are consistent with the phenotypic observations, which showed no differences in Col-0 and *PUB13*/*pub13*, while both *pub13* and *PUB13^V273R^/pub13* exhibited relatively low values (**Supplementary Figure S1**). These results suggest that the E3 ubiquitin ligase activity of PUB13 is required for plant growth.

### 3.2 PUB13 negatively regulates flowering time depending on the E3 ubiquitin ligase activity

**Figure 2:**
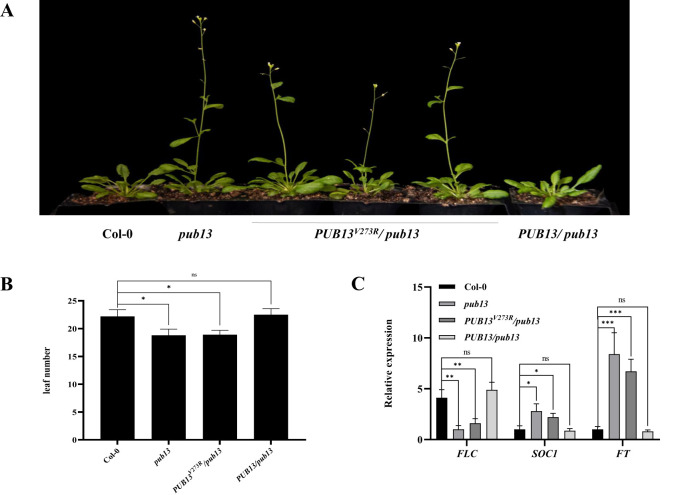
The E3 Ubiquitin Ligase Activity of PUB13 Is Required for Regulating Flowering Time. **(A)** Flowering phenotypes of Col-0, *pub13*, *PUB13^V273R^/ pub13*, and *PUB13/ pub13* grown under long day conditions. **(B)** The rosette leaves number of indicated plants. The leaves number was counted when the first flower bud appeared. **(C)**, Expression of flowering marker genes *FLC*, *SOC1*, and *FT* in indicated plants. *Actin* was used internal reference. Asterisks denote significant difference based on nested ANOVA (*P < 0.05, **P < 0.01, ***P < 0.001). These experiments were repeated three times with similar results.

Our previous results showed that PUB13 negatively regulates flowering time in *Arabidopsis* (Li et al., 2012a). We also examined whether the E3 ubiquitin ligase activity of PUB13 is required for the regulation of *Arabidopsis* flowering time. The *pub13* mutant showed an early flowering phenotype under long-day conditions, as reported, while the complementary plants *PUB13/pub13* did not show early flowering (**Figure 2A**). However, the *PUB13^V273R^/pub13* plants also showed a noticeable early flowering phenotype, similar to *pub13* plants (**Figure 2A**). Correspondingly, the number of rosette leaves before flowering of the *pub13* and *PUB13^V273R^/pub13* plants was significantly lower than that of Col-0, while the number of rosette leaves of *PUB13/pub13* did not show significant differences compared with Col-0 (**Figure 2B**). To confirm the function of PUB13 enzyme activity on flowering, the negative flowering regulator *FLC* and the positive regulators *FT* and *SOC1* in the above plants were detected using RT-qPCR. The results showed that the expression of *FLC* was suppressed in *pub13* and *PUB13^V273R^/pub13* compared with that in Col-0 (**Figure 2C**). In contrast, the expression of *FT* and *SOC1* in *pub13* and *PUB13^V273R^/pub13* was enhanced compared with that in Col-0, and the expression of all three genes did not show significant differences between Col-0 and *PUB13/pub13* (**Figure 2C**). Therefore, the results of the expression of the flowering regulator genes in the above plants are consistent with the flowering phenotypes in **Figure 2A**. Taken together, PUB13 regulates flowering time depending on its E3 ubiquitin ligase activity.

### 3.3 E3 ubiquitin ligase activity is required for PUB13 regulating disease resistance

**Figure 3:**
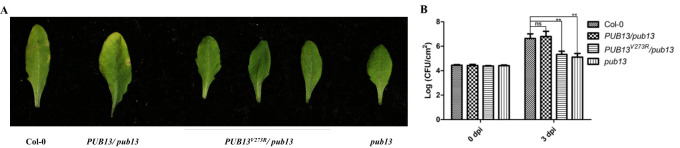
E3 Ubiquitin Ligase Activity Is Required for PUB13 Regulated Disease Resistance. **(A)** Disease symptoms of Col-0, *PUB13/pub13*, *PUB13^V273R^/pub13* and *pub13* inoculated with *Psm* ES4326. Four weeks old plants growing under long day conditions were sprayed with 1×10^8^ CFU mL^-1^ of *Psm* ES4326, and observed the symptoms at 3 dpi. **(B)** Bacterial growth in Col-0, *PUB13/pub13*, *PUB13^V273R^/pub13* and *pub13* inoculated with *Psm* ES4326. Four weeks old plants growing under long day conditions were injected with 5×10^5^ CFU mL^-1^ *Psm* ES4326. The bacteria numbers in injected leaves were counted at 0 dpi and 3 dpi, respectively. Asterisks denote significant difference based on nested ANOVA (*P < 0.05, **P < 0.01). These experiments were repeated at least three times with similar results.

As PUB13 regulates plant disease resistance based on our previous research (Li et al., 2012a), we determined whether E3 ubiquitin ligase activity is required for PUB13-regulated resistance. The Col-0, *PUB13/pub13*, *PUB13^V273R^/pub13*, and *pub13* plants were inoculated with the bacterial pathogen *P. syringae* pv. *maculicola* ES4326. After 3 days post-inoculation (dpi), the leaves of both Col-0 and *PUB13/pub13* showed leaf chlorosis, while the leaves of *pub13* appeared healthy. *PUB13^V273R^/pub13* was as resistant as *pub13* (**Figure 3A**), which indicates that the E3 ubiquitin ligase activity-suppressed PUB13^V273R^ cannot complement *pub13* to the wild type. Furthermore, the bacterial growth in the above plants was assessed after infiltration with *Psm* ES4326 at a lower concentration. The results showed that the susceptibility of *PUB13/pub13* recovered to that of the wild-type Col-0, while the resistance of *PUB13/pub13* recovered to the wild-type level (**Figure 3B**). Taken together, the pathogen inoculation results demonstrated that E3 ubiquitin ligase activity is required for PUB13 negatively regulating plant resistance against bacterial pathogens.

### 3.4 PUB13 interacts with and ubiquitinates BON1

**Figure 4:**
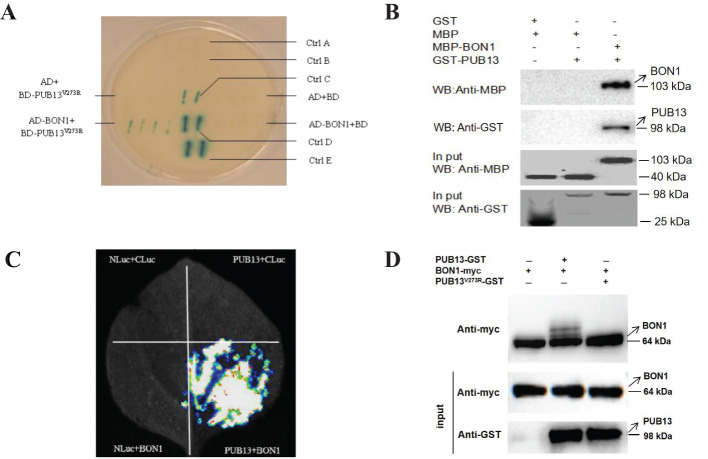
PUB13 Interacts with and Ubiquitinates BON1. **(A)** PUB13^V273R^ interacted with BON1 in yeast. The full-length CDS of *PUB13^V273R^* and *BON1* were cloned into pDBleu (BD) and pPC86 (AD), respectively, then transformed them into yeast strain MaV203. The yeast transformants were plated on YPDA medium for X-gal assay. Ctrl (A-E) represent A: pDEST™32; B: pDEST™22; C: pEXP™32/Krev1+pEXP™22/RalGDS-m1; D: Bait plasmid+Known prey; E: pEXP™32/Krev1+pEXP™22/RalGDS-wt. These five controls indicate the spectrum of interaction strengths. **(B)**, The GST pull-down assay confirmed the interaction between BON1 and PUB13. MBP-BON1 and GST-PUB13 proteins were mixed and conducted GST pull down, then detected the eluted protein by Western blotting with the anti-MBP antibody; **(C)**, LCI assay in *N. benthamiana* leaves proved the interaction between PUB13 and BON1. Each of the quadrants was infiltrated with PUB13-Nluc or BON1-Cluc and the indicated construct. **(D)**, PUB13 ubiquitinates BON1. The upper panel is anti-myc WB to detecte BON1 treated with E1, E2, and indicated proteins, the middle panel is anti-myc WB to detect BON1 before treating with E1 and E2 as loading control, and the bottom panel is anti-GST WB to detect PUB13 and PUB13^V273R^. The ubiquitination of BON1-myc by PUB13-GST and PUB13^V273R^-GST in the presence of E1, E2. Ubiquitination was detected with anti-myc Western-blot.

It has been reported that PUB13 interacts with BAK1, HFR1, and ABI1 to regulate plant immunity (Li et al., 2009; Lu et al., 2011; Li et al., 2021). To further illustrate the molecular mechanism of PUB13 regulating growth, development, and immunity, PUB13^V273R^, which cannot degrade target proteins, was used as bait for the yeast two-hybrid assay to screen its interacting proteins (**Supplementary Table S1**). Among the screened interactors, the calcium-dependent phospholipid-binding protein BON1 was studied as it is involved in plant growth homeostasis and disease resistance. Firstly, we confirmed the interaction between BON1 and PUB13^V273R^ in the yeast system (**Figure 4A**). Secondly, the GST pull-down experiment was conducted, which showed that PUB13 interacted with BON1 *in vitro* (**Figure 4B**). Lastly, the interaction between PUB13 and BON1 was proven through luciferase complementation imaging (LCI) experiment on *N. benthamiana* (**Figure 4C**). The results of these assays indicate that PUB13 physically interacts with BON1.

In consideration of PUB13 functioning as an E3 ubiquitin ligase, we determined whether PUB13 and the enzyme inactive mutant PUB13^V273R^ ubiquitinate the interactor BON1. According to the ubiquitination assay, *in vitro*, PUB13 ubiquitinated BON1 without obvious degradation in the presence of the ubiquitin-activating enzyme E1, the ubiquitin-conjugating enzyme E2, and ubiquitin, while PUB13^V273R^ did not ubiquitinate BON1 in the same reaction conditions (**Figure 4D**).

### 3.5 BON1 synergizes with PUB13 to regulate plant growth

**Figure 5:**
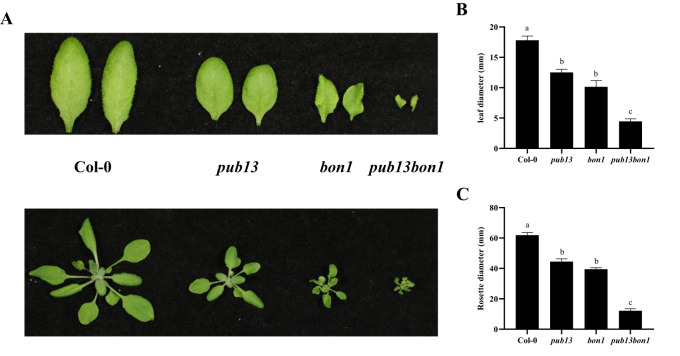
BON1 Synergizes with PUB13 to Regulate Plant Growth. **(A)**, The leaves and plants of Col-0, *pub13*, *bon1*, and *pub13*(-/-)*bon1*(+/-) under long day conditions. **(B)**, The statistics of leaf width diameters of Col-0, *pub13*, *bon1*, and *pub13*(-/-)*bon1*(+/-). **(C)**, The rosette diameters of Col-0, *pub13*, *bon1*, and *pub13*(-/-)*bon1*(+/-). Lowercase letters indicate significant difference at P<0.01. All experiments were repeated at least three times with similar results.

As previously reported, PUB13 regulates plant growth depending on its E3 ubiquitin ligase activity (12). Similarly, the *bon1* mutant also showed a growth defect phenotype, such as leaf shrinkage and plant dwarfing (**Figure 1**). To analyze the interaction between BON1 and PUB13 on the regulation of physiological functions, we constructed *bon1* and heterozygous *pub13(−/−)bon1(+/−)* mutants, with the homozygous *pub13(−/−)bon1(−/−)* mutant being lethal. As expected, both the *bon1* and *pub13* plants, including their leaves, were smaller than the Col-0 plants (**Figure 5A**). Furthermore, the *pub13(−/−)bon1(+/−)* plants, as well as their leaves, were even much smaller than *pub13* and *bon1* (**Figure 5A**). The statistics for the leaf size and rosette diameters of the above plants were consistent with the above plant growth phenotypes (**Figures 5B, C**), indicating that BON1 synergized with PUB13 to regulate plant growth.

### 3.6 BON1 negatively regulates flowering time

**Figure 6:**
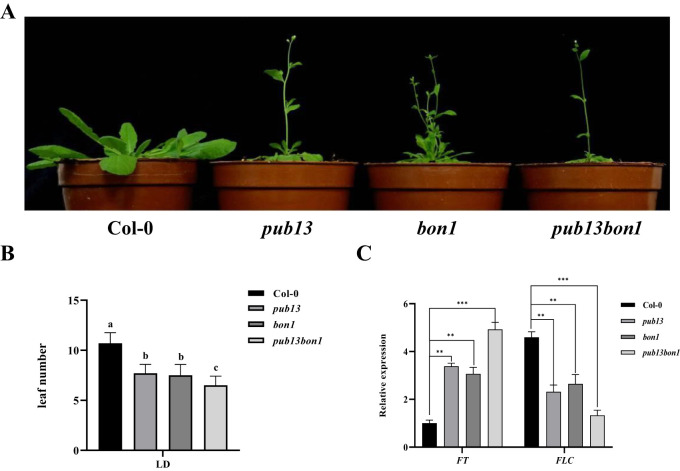
BON1 Negatively Regulates Flowering Time. **(A)**, Flowering phenotypes of *pub13*, *bon1*, and *pub13*(-/-)*bon1*(+/-). Plants were grown under long day conditions. **(B)**, The leaf numbers of Col-0, *pub13*, *bon1*, and *pub13*(-/-)*bon1*(+/-) plants before flowering under long day conditions. Lowercase letters indicate significant difference at P<0.01. **(C)**, Expression of flowering marker genes in Col-0、*pub13*、*bon1* and *pub13*(-/-)*bon1*(+/-) under long day conditions. Gene expression of *FLC* and *FT* in indicated plants was determined by qRT-PCR. *Actin* was used as internal reference. Asterisks denote significant difference based on nested ANOVA (**P < 0.01, ***P < 0.001). All experiments were repeated at least three times with similar results.

PUB13 negatively regulates flowering time, particularly under long-day conditions (Li et al., 2012a). Therefore, the function of BON1 on flowering time was analyzed. The heterozygous *pub13(−/−)bon1(+/−)*, as well as Col-0, *pub13*, and *bon1*, was planted under long-day conditions. As expected, the *pub13* mutant showed an early flowering phenotype compared with Col-0 (**Figure 6A**). Interestingly, both the *bon1* and *pub13(−/−)bon1(+/−)* mutants showed early flowering, while *pub13(−/−)bon1(+/−)* did not show a noticeably earlier flowering compared with the *bon1* and *pub13* single mutants (**Figure 6A**). To confirm the flowering phenotypes, the leaf number of the above plants was counted before flowering. The results showed that the rosette leaf numbers of *pub13* and *bon1* were significantly fewer than that of Col-0, while that of *pub13(−/−)bon1(+/−)* was extremely significantly fewer than that of Col-0 (**Figure 6B**). Moreover, the transcription levels of the flowering marker genes *FT* and *FLC* in these plants were examined with RT-qPCR. As shown in **Figure 6C**, the transcription levels of the negative flowering regulator *FLC* in *pub13*, *bon1*, and *pub13(−/−)bon1(+/−)* were noticeably lower than that in Col-0. In contrast, the expression of the positive flowering regulator *FT* was significantly higher in *pub13*, *bon1*, and *pub13(−/−)bon1(+/−)* compared with Col-0 (**Figure 6C**). These data indicate that BON1 negatively regulates flowering time, probably through a PUB13 independent pathway.

### 3.7 BON1 synergizes with PUB13 to negatively regulate resistance to biotrophic pathogens

**Figure 7:**
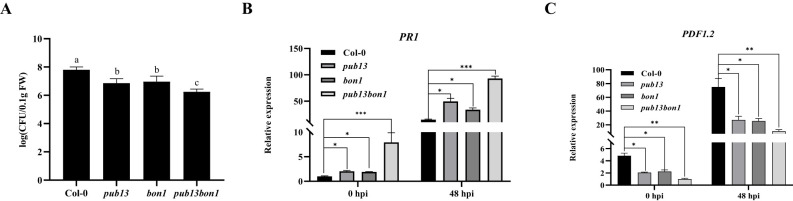
BON1 Synergizes with PUB13 to Negatively Regulate Resistance to *Pst* DC3000. **(A)**, Bacterial growth of *Pst* DC3000 in Col-0, *pub13*, *bon1*, and *pub13*(-/-)*bon1*(+/-) at 48 hpi. The leaves were injected with 5×10^5^ CFU mL^-1^ *Pst* DC3000.Lowercase letters indicate significant difference at P<0.01. **(B, C)**, The transcription level of *PR1* and *PDF1.2* in plants inoculated with DC3000. After injected the indicated plants with *Pst* DC3000, the RNA was extracted at 0 hpi and 48 hpi for qRT-PCR. *Actin* was used as internal reference. Asterisks denote significant difference based on nested ANOVA (*P < 0.05, **P < 0.01, ***P < 0.001). These experiments were repeated three times with similar results.

Considering PUB13 negatively regulating plant resistance to biotrophic pathogens, the role of BON1 in PUB13-regulated disease resistance was analyzed. Col-0, *pub13*, *bon1*, and *pub13(−/−)bon1(+/−)* plants were inoculated with *P. syringae* pv. *tomato* DC3000, which is a biotrophic pathogen at the early stage of infection and mainly defended by the salicylic acid (SA) resistance pathway. The bacterial growth results showed that both the *pub13* and *bon1* mutants were more resistant than Col-0. Furthermore, *pub13(−/−)bon1(+/−)* was found to be even more resistant than *pub13* and *bon1* (**Figure 7A**). We further analyzed the expression of the defense-related genes *PR1* and *PDF1.2* in the above plants before and after pathogen inoculation. At 0 and 48 h post-infection (hpi), the expression of the SA defense pathway gene *PR1* in the *pub13* and *bon1* mutants was increased, and *PR1* in *pub13bon1* was higher than that in *pub13* and *bon1* (**Figure 7B**). In contrast, the expression of the *PDF1.2* gene in the jasmonic acid (JA)/ethylene (ET) defense pathway, which is antagonistic to the SA defense pathway, was inhibited in *pub13* and *bon1* and was lowest in *pub13(−/−)bon1(+/−)* (**Figure 7C**). These results indicate that BON1 synergizes with PUB13 to negatively regulate resistance to *Pst* DC3000.

### 3.8 Knockout of BON1 increases PUB13 susceptibility to necrotrophic pathogens

**Figure 8:**
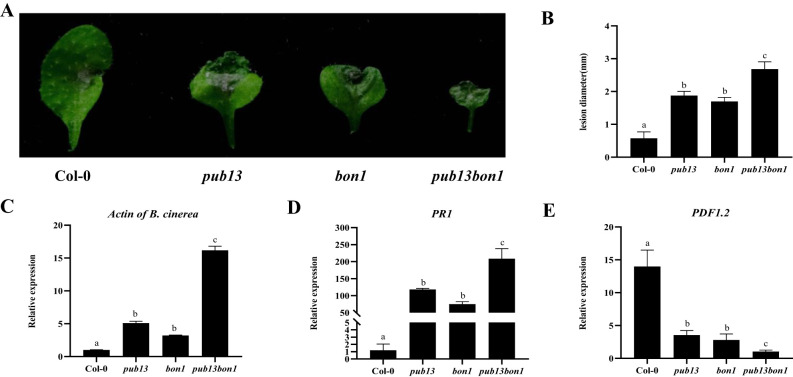
BON1 Synergizes with PUB13 to Positively Regulate Resistance to to *Botrytis cinerea.* **(A)**, Disease symptoms of Col-0, *pub13*, bon*1*, and *pub13*(-/-)*bon1*(+/-) infected with *B. cinerea* BO5-10. Detached leaves were inoculated with 5 µL of conidia suspension (3×10^4^ conidia mL^-1^) of BO5-10, then kept the samples under high humidity conditions, and observe the symptoms at 48 hpi. B, Lesion size of plants inoculated with **(*B*)** *cinerea* BO5-10 at 48 hpi. **(C-E)**, Transcription level of BO5-10 *Actin*, *PR1* and *PDF1.2* in indicated plants after inoculation with *Botrytis cinerea* BO5-10 at 48 hpi. Arabidopsis *Actin* was used as internal control. Lowercase letters indicate significant difference at P<0.01. All experiments were repeated at least three times with similar results.

To determine the function of BON1 in the PUB13-regulated defense to necrotrophic pathogens, Col-0, *pub13*, *bon1*, and *pub13(−/−)bon1(+/−)* plants were inoculated with the necrotrophic pathogen *B. cinerea* BO5-10, which is mainly defended by the JA/ET resistance pathway. According to the observed symptoms, both *pub13* and *bon1* were more susceptible than Col-0, and *pub13(−/−)bon1(+/−)* was even more susceptible than *pub13* and *bon1* (**Figure 8A**). Correspondingly, the lesion diameters of *pub13* and *bon1* were significantly bigger than that of Col-0, with that of *pub13(−/−)bon1(+/−)* being the biggest (**Figure 8B**). The expression of *B. cinerea Actin*, which represents fungal biomass, in *pub13* and *bon1* was higher than that in Col-0 at 48 hpi and was highest in *pub13(−/−)bon1(+/−)* (**Figure 8C**). In addition, the transcription level of *PR1* in these four plants at 48 hpi showed the same trend as that of *B. cinerea Actin* (**Figure 8D**). However, the expression of *PDF1.2* was opposite to that of *PR1*, where *PDF1.2* was inhibited in *pub13* and *bon1* at 48 hpi and was even lower in *pub13(−/−)bon1(+/−)* than in *pub13* and *bon1* (**Figure 8E**). These results further indicate that BON1 synergizes with PUB13 to positively regulate plant resistance to necrotrophic pathogens.

### 3.9 BON1 is involved in the PTI response regulated by PUB13

**Figure 9:**
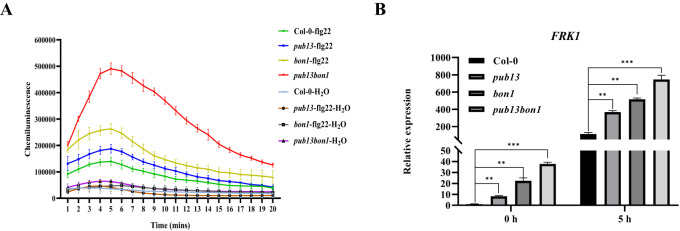
PUB13 Synergizes with BON1 to regulate PTI. **(A)**, flg22 induced ROS accumulation in Col-0, *pub13*, *bon1*, and *pub13*(-/-)*bon1*(+/-) leaves. ROS was determined using the luminol-based chemiluminescence. Three leaf discs for each sample were treated with 100 nM flg22 or sterilized distilled water as control. **(B)**, *FRK1* expression in Col-0, *pub13*, *bon1*, and *pub13*(-/-)*bon1*(+/-) upon flg22 induction. After injecting 100 nM *flg22*, RNA was extracted at 0 h and 5h for RT-qPCR. *Actin* was used as internal reference. Asterisks denote significant difference based on nested ANOVA (**P < 0.01, ***P < 0.001). These experiments were repeated three times with similar results.

To determine whether BON1 is involved in PUB13-regulated PTI signaling, the PAMP flg22-induced PTI responses were investigated in Col-0, *pub13*, *bon1*, and *pub13(−/−)bon1(+/−)* plants. Upon stimulation with flg22, the accumulation of ROS was enhanced in these plants (**Figure 9A**). Notably, *pub13* and *bon1* showed stronger induction of flg22-triggered ROS compared with Col-0, and the *pub13(−/−)bon1(+/−)* double mutant showed more ROS accumulation than the other three plants, indicating additive effects between PUB13 and BON1 on PTI response (**Figure 9A**). Furthermore, the expression of the marker gene *FRK1* in the PTI signaling pathway was examined by RT-qPCR. The results showed that before (0 hpi) or after (5 hpi) flg22 treatment, the *FRK1* expression in *pub13*, *bon1*, and *pub13(−/−)bon1(+/−)* was significantly upregulated compared with Col-0 and was highest in *pub13(−/−)bon1(+/−)* (**Figure 9B**). These data suggest that both PUB13 and BON1 function as negative regulators of PTI and that BON1 is involved in the PTI response regulated by PUB13.

## 4 Discussion

The *Arabidopsis* E3 ubiquitin ligase PUB13, as an important hub of several signals, regulates multiple plant biological processes, including growth, flowering, plant immunity, and cell death (Lu et al., 2011; Li et al., 2012a; Kong et al., 2015). In this study, it was found that PUB13 regulated plant growth, flowering time, and disease resistance. On the other hand, the copine protein BON1 was identified as an interactor and a ubiquitination substrate of PUB13, which enhanced its biological functions on growth, flowering, and immunity. Moreover, BON1 can be ubiquitinated, but not degraded, by PUB13. Here, we hypothesize that PUB13 activates BON1 through ubiquitination, enabling their cooperative regulation of plant stress responses through synergistic signaling.

On the other hand, PUB13 negatively regulated the plant flowering time and disease resistance depending on the SA and JA/ET signaling pathways (Li et al., 2012a). Based on the enhanced *PR1* and the inhibited *PDF1.2* expression in *bon1* and *pub13(−/−)bon1(+/−)*, conceivably, BON1 synergizes with PUB13 to negatively regulate disease resistance probably via the SA and JA/ET signaling pathways. SA and JA/ET are major phytohormones that mediate plant disease resistance against biotrophic and necrotrophic pathogens, respectively (Shigenaga et al., 2017; Costarelli et al., 2020). The increase in the transcription levels of *PR1* indicates that the SA signaling pathway was activated in *bon1* and *pub13(−/−)bon1(+/−)* mutants, as well as in *pub13*, leading to the enhanced resistance to biotrophic pathogens regulated by the SA signaling pathway. In contrast, the decrease in the transcription levels of *PDF1.2* suggests that the JA/ET signaling pathway was suppressed in the *pub13*, *bon1*, and *pub13(−/−)bon1(+/−)* mutants, resulting in the increased susceptibility to necrotrophic pathogens regulated by the JA/ET signaling pathway.

Both PUB13 and BON1 simultaneously regulate plant disease resistance and growth, indicating the correlation between these two biological processes. According to numerous studies, the enhancement of disease resistance usually leads to the inhibition of plant growth by the resistance substances in the plant; hence, there is a certain antagonistic relationship between these two biological processes, which is a challenge in the field of disease resistance breeding. For example, *OsBON1* or *OsBON3*, the orthologs of *Arabidopsis* BON1, suppresses disease resistance, but promotes plant growth (Yin et al., 2018). Moreover, a pair of homologous E3 ubiquitin ligases (APIP6 and IPI1) in rice enhances the resistance to rice blast by facilitating the degradation of the circadian clock-related protein OsELF3 (Xu et al., 2024). In addition, the OsELF3 protein is associated with the regulation of flowering time in rice; therefore, its degradation results in a delay in the flowering period of rice (Xu et al., 2024). On the other hand, the *pub13* and *bon1* mutants displayed inhibited growth, but they were early flowering, implying the phenomenon that plants will start their development stage in advance once their growth is inhibited, which is a self-preservation mechanism in plants.

In this study, it was found that the ubiquitination system protein PUB13 interacts with the Ca^2+^-dependent phospholipid-binding protein BON1 and that they can synergistically regulate the growth, development, and disease resistance in *Arabidopsis*. The interaction between calcium signaling and the ubiquitination pathway has been reported (Mukherjee et al., 2017). The ubiquitination pathway can regulate the stability and activity of proteins involved in calcium signaling transduction, thus influencing the transmission and response of calcium signals (Meng et al., 1999; Wang et al., 2010). For example, the calmodulin-regulated transcription factor AtSR1/CAMTA3 in *Arabidopsis* negatively regulates the SA-regulated plant disease resistance according to the ubiquitination system (Zhang et al., 2014). When the plant is invaded by pathogens, the ubiquitination and the degradation of AtSR1 are activated, leading to the suppression of the AtSR1-regulated calcium/calmodulin signaling, which results in the SA-mediated plant defense being enhanced (Zhang et al., 2014). Nevertheless, in-depth research is needed on the crosstalk between plant disease resistance and growth/development, as well as on the interaction mechanism between ubiquitination and calcium signaling in plant immune responses, which will provide a better understanding of the signal transduction network of plant disease resistance.

## Data Availability

The original contributions presented in the study are included in the article/supplementary material. Further inquiries can be directed to the corresponding authors.

## Supplementary material

Supplementary Table 1 Part of library screening results by yeast two-hybrid.
