# Supplementary material for: The Arabidopsis E3 ubiquitin ligase PUB13 synergistically interacts with BON1 to regulate plant flowering and immunity
Source: Front Plant Sci. 2025 Jun 2;16:1585221. doi: 10.3389/fpls.2025.1585221 (PMC12171143; doi:10.3389/fpls.2025.1585221)
Supplement: Supplementary file 2 [file Table2.docx]

**Supplemental Figure 1 The growth parameters of Col-0, *pub13*, *PUB13^V273R^/ pub13*, and *PUB13/ pub13***

A, The rosette diameters of Col-0, *pub13*, *PUB13^V273R^/ pub13*, and *PUB13/ pub13*. B, The statistics of leaf petiole length of Col-0, *pub13*, *PUB13^V273R^/ pub13*, and *PUB13/ pub13*. B, The statistics of leaf lamina length of Col-0, *pub13*, *PUB13^V273R^/ pub13*, and *PUB13/ pub13*. Lowercase letters indicate significant difference at P<0.01. All experiments were repeated at least three times with similar results.
